# Supplementary material for: Increased localization of APP‐C99 in mitochondria‐associated ER membranes causes mitochondrial dysfunction in Alzheimer disease
Source: EMBO J. 2017 Oct 10;36(22):3356–71. doi: 10.15252/embj.201796797 (PMC5731665; doi:10.15252/embj.201796797)
Supplement: Supplementary file 1 — Appendix [file EMBJ-36-3356-s001.pdf]

Appendix:

Increased localization of APP-C99 in mitochondria-associated ER membranes causes mitochondrial dysfunction in Alzheimer disease

Supplementary Information:

Appendix figure S1
Appendix figure S2
Appendix figure S3
Appendix figure S4
Appendix figure S5
Appendix figure S6
Appendix figure S7

Appendix Figure S1

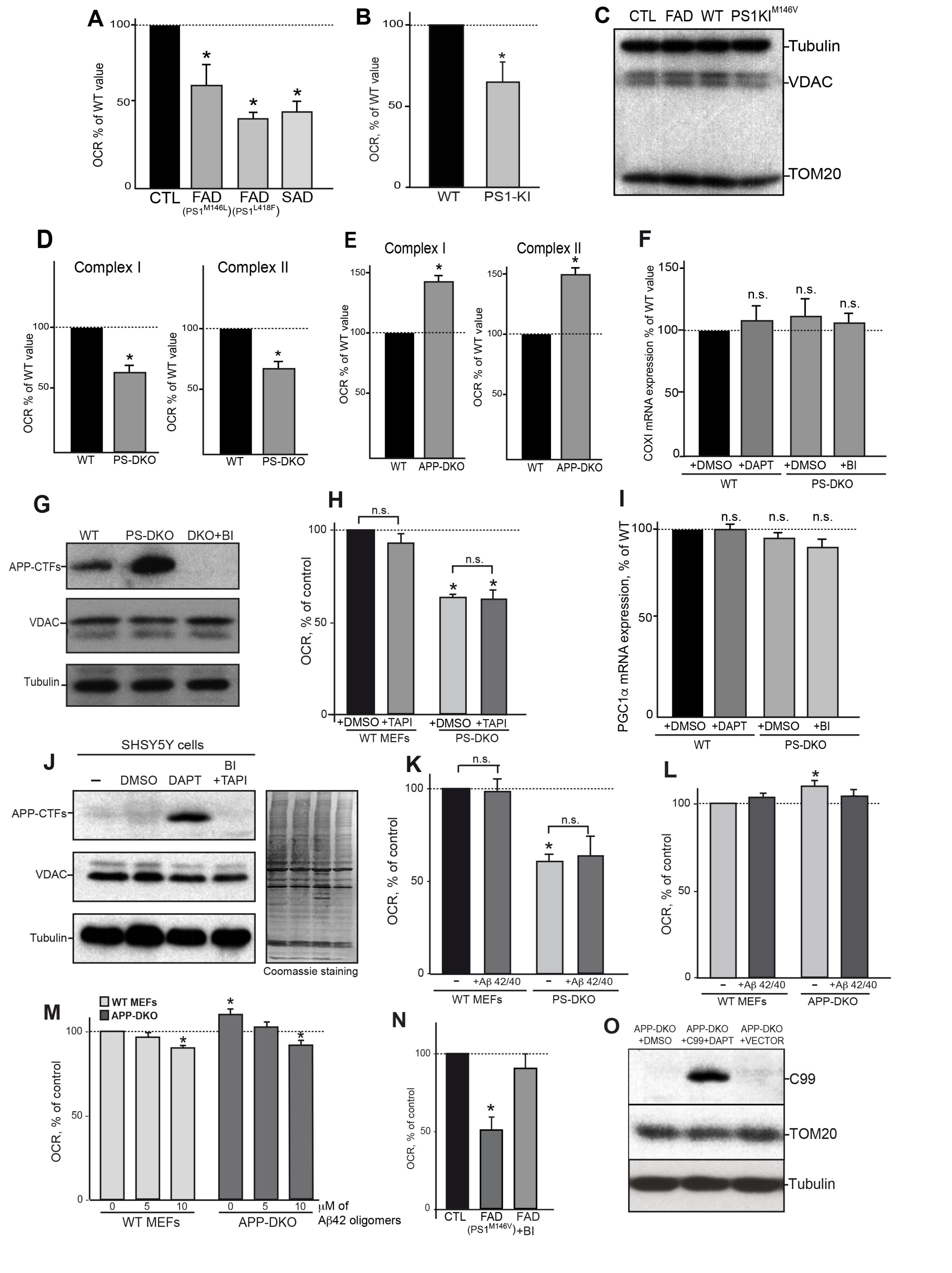

Appendix Figure S1 (cont.)

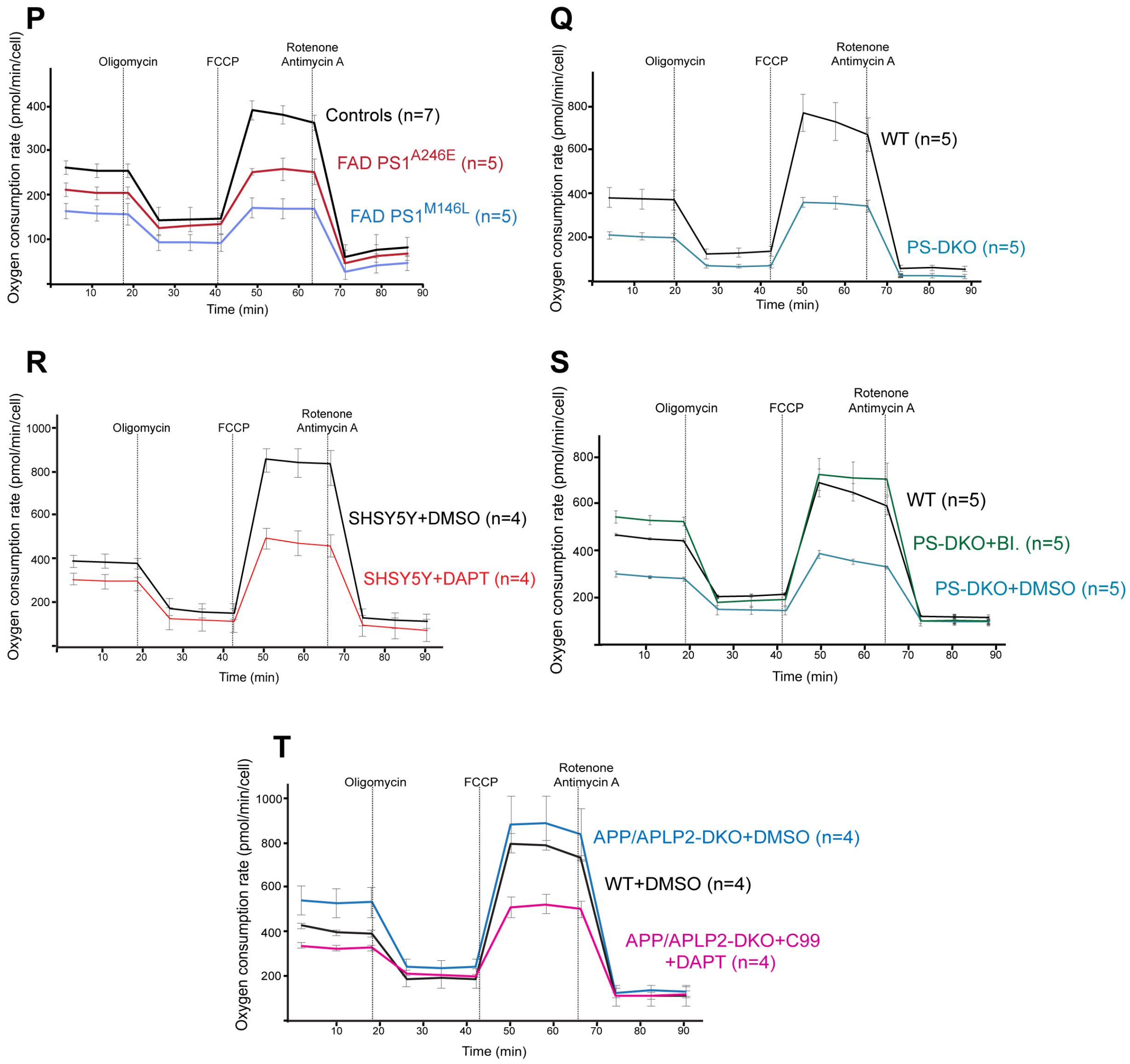

Appendix Figure S1. Mitochondrial bioenergetics in γ-secretase-deficient cells. All respiratory chain assays (OCR) were performed using the Seahorse XF24 Flux Analyzer. **(A)** Fibroblasts from FAD and SAD patients, **(B)** Mitochondria from PS1-KI^{M146V} brain. **(C)** Western blot from homogenates from the indicated cells probed against mitochondrial markers (VDAC and TOM20) and the tubulin loading controls (30 μg of protein per lane). **(D, E)** Complex I and

II activities in which malate-pyruvate or succinate-rotenone, respectively, were added to permeabilized PS-DKO MEFs (D) and to APP-DKO MEFs (E). Note significant decrease in OCR in PS-DKO MEFs but an increase in APP-DKO MEFs. **(F)** qRT-PCR to measure mRNA levels of mitochondrial encoded *Cox1* as a measure of mitochondrial DNA in WT and PS-DKO cells before and after addition of DAPT and BI. n.s.=not significant. **(G)** Western blot from total homogenates of WT, PS-DKO, and PS-DKO cells treated with BACE1 inhibitor (BI); 30 μg of protein per lane). Note that BI treatment eliminates the accumulation of APP C-terminal fragments in PS-DKO MEFs, without changes in mitochondria (VDAC, TOM20). **(H)** OCR in WT or PS-DKO cells is unaffected by treatment with the α-secretase inhibitor TAPI-1. **(I)** qRT-PCR to measure mRNA levels of PGC-1α, a master regulator of mitochondrial biogenesis, in WT and PS-DKO cells before and after addition of DAPT and BI (which do not affect mitochondrial biogenesis). Note lack of an effect on PGC-1α levels in γ-secretase-deficient cells. n.s., not significant. **(J)** Western blot from total homogenates of SHSY5Y cells treated with DMSO and α-, β-, and γ-secretase inhibitors probed with the indicated antibodies. Note that none of the treatments change the levels of mitochondria (VDAC). Loading control in the right panel (30 μg of protein per lane). **(K, L)** Addition of monomers of $A\beta_{40}$ and $A\beta_{42}$ (added at a ratio of 10:1 $A\beta_{40}$:$A\beta_{42}$, total concentration of Aβ was 6 ng/ml) to PS-KO cells (K), or to APP-DKO cells (L) did not affect mitochondria respiration. **(M)** Addition of 0, 5, and 10 μM of $A\beta_{42}$ oligomers to WT and APP-DKO cells shows that only at a high-non-physiological concentration (10 μM) of $A\beta_{42}$ oligomers, mitochondrial respiration is slightly decreased. **(N)** Respiratory chain deficiency in fibroblasts from an FAD patient (AG06840. $PS1^{A246E}$) was rescued following treatment with a BACE1 inhibitor. **(O)** Western blot showing how transient transfection of C99 in APP-DKO cells did not affect mitochondrial levels (TOM20). Tubulin is revealed as loading controls. All western blots were performed on the same membranes consecutively. **(P-T)** Seahorse graphs of representative experiments shown in Figure 1. All experiments represent the average of n≥4 independent experiments; n.s., non significant; 30 μg of protein per lane.

Appendix Figure S2

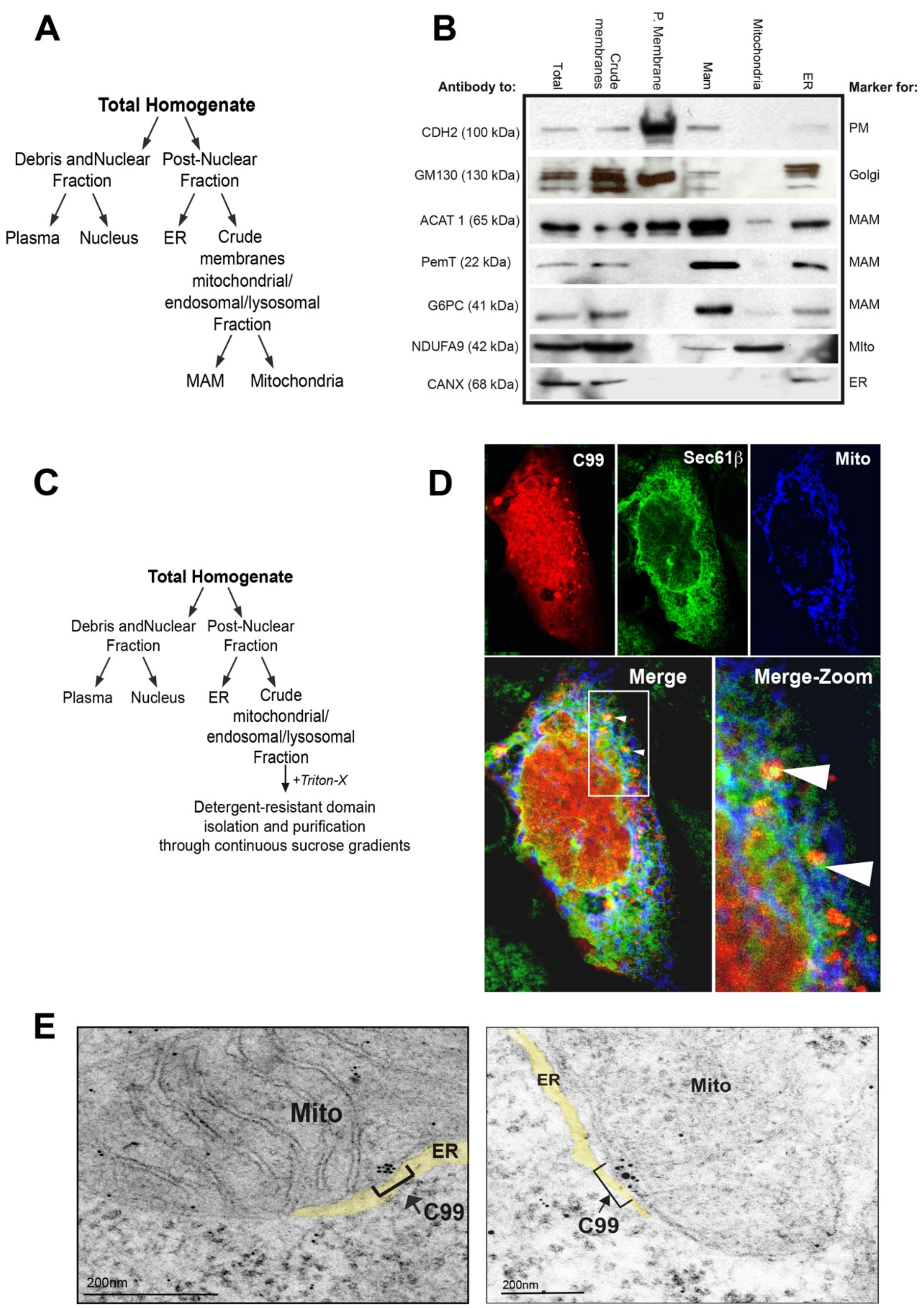

Appendix Figure S2 (Cont.)

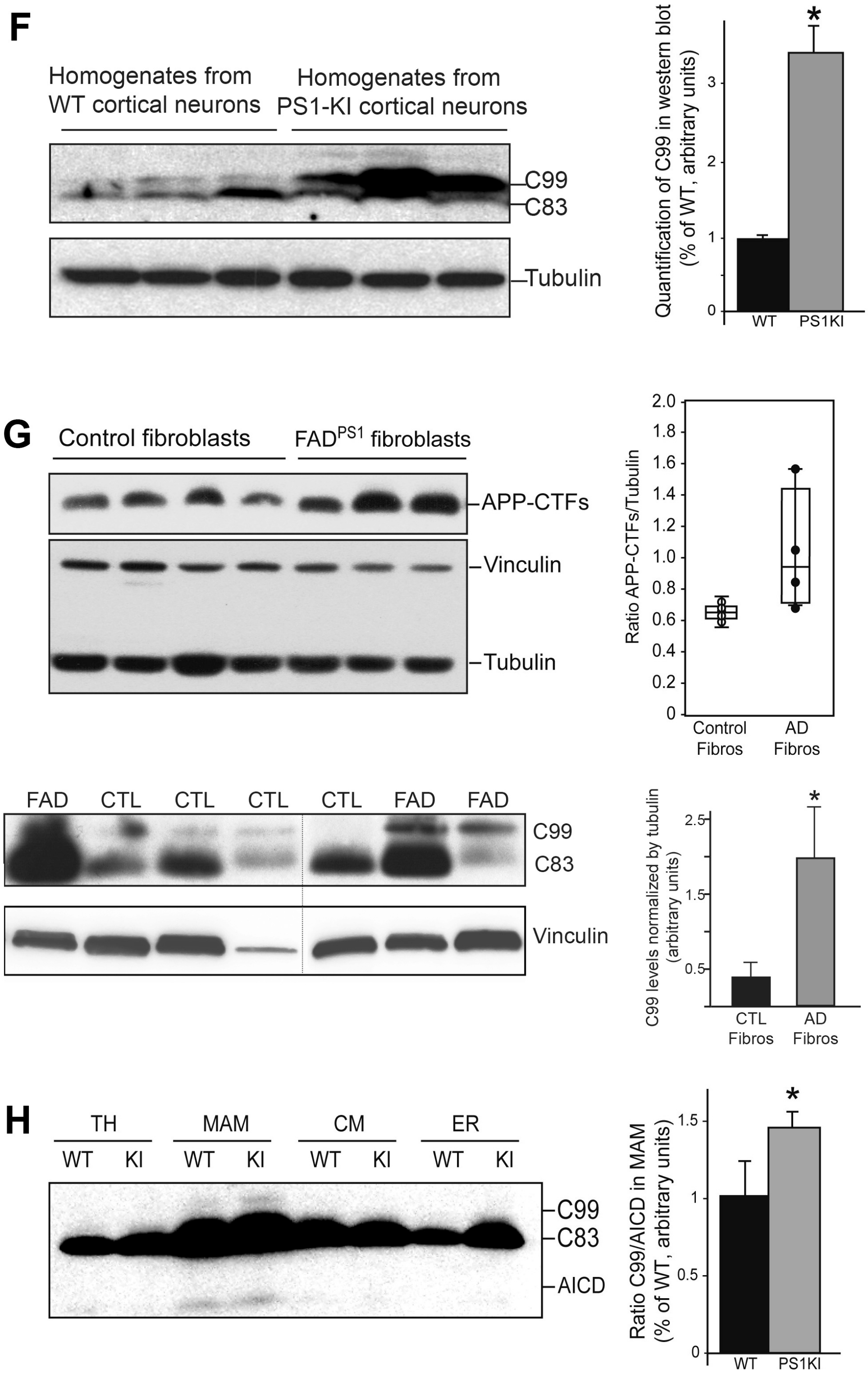

Appendix Figure S2. Localization of C99 to MAM in PS1-KI^{M146V} mice. (A) Scheme showing the protocol followed to isolate the subcellular fractions analyzed by Western blot. **(B)** Example of western blot analysis of subcellular fractions from mouse liver obtained using the protocol shown in panel S2A, probed against specific markers for each indicated compartment (30 μg of protein per lane). **(C)** Scheme showing the protocol followed to purify subcellular fractions by continuous sucrose gradients analyzed by Western blot as shown in Figure 2C. **(D)** Representative confocal images from WT MEFs non-treated with DAPT and transfected with C99 (red), Sec61β to label ER (green), and mitochondria (blue). White arrowheads indicate areas of contact between ER and mitochondria where C99 is present. Red signal in the nucleus corresponds to the APP intracellular domain (AICD) produced after the γ-secretase cleavage of the transfected C99-red construct (compare to DAPT-treated COS-7 cells in Figure 2E, where no AICD is produced after γ-secretase inhibition). **(E)** Representative electron microscopy images of PS-DKO cells incubated with antibodies against APP-CTF conjugated with immunogold particles show significant labeling in MAM regions (brackets). **(F)** Western blots of total homogenates of embryonic cortical neurons explanted from WT and PS1-KI^{M146V} mice, and of fibroblasts from AD patients (**G**) show higher levels of C99 in mutant samples vs. controls (30 μg of protein per lane). All samples were run on the sample gel. Dotted line indicates an empty lane cut out. Quantifications of western blot bands normalized to the loading controls (tubulin and vinculin) are shown in the panels at right. **(H)** Western blot of subcellular fractions isolated from WT and PS1-KI^{M146V} shows increased levels of C99 in total homogenates (TH), crude membranes (CM), ER, and MAM fractions. Note that AICD levels are not significantly changed compared to C99 (quantification in the panel at right) (Western blots of total homogenates of embryonic cortical neurons explanted from WT and PS1-KI^{M146V} mice, and of fibroblasts from AD patients (**G**) show higher levels of C99 in mutant samples vs. controls (30 μg of protein per lane). Quantifications of western blot bands normalized to the loading controls (tubulin and vinculin) are shown in the panels at right.

Appendix Figure S3

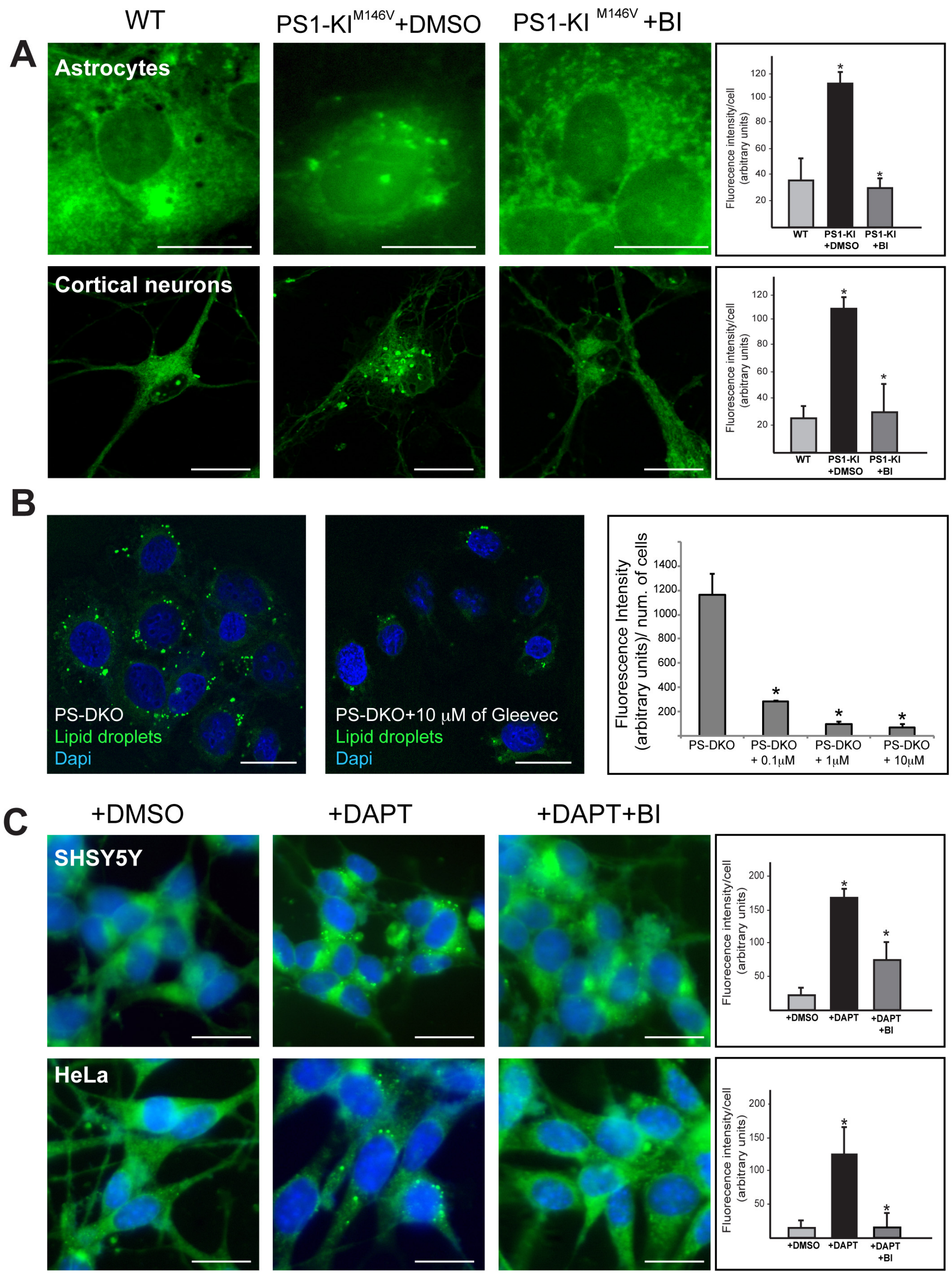

D

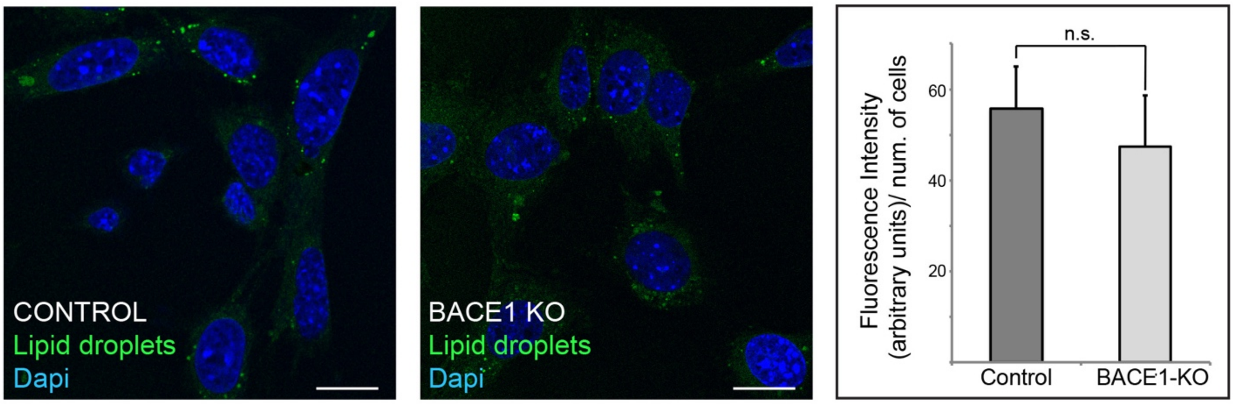

Appendix Figure S3. Cholesteryl ester and lipid droplet formation in γ-secretase-deficient cells. (A) Staining with LipidTox Green to detect lipid droplets in the indicated explanted astrocytes (size bar=20 µm) and cortical neurons (size bar=25 µm) from WT and PS1-KI^{M146V} mice. **(B)** Staining of LDs as in (A) of PS-DKO cells untreated and treated with 10 µM of Gleevec. Quantitation of lipid droplets after incubation of PS-DKO cells with increasing concentrations of Gleevec is shown at right. All values represent the average of n=3 independent experiments ± SD; * p<0.01. **(C)** Staining of SH-SY5Y cells (upper panels) or HeLa cells (lower panels) treated with DAPT (to increase C99 accumulation) or with DAPT+BI (to inhibit C99 production) (size bar=20µm). Quantitation of lipid droplets is shown at right. All values represent the average of n=3 independent experiments ± SD; * p<0.01. **(D)** Staining of BACE1-KO cells. Quantitation of lipid droplets is shown at right. All values represent the average of n=3 independent experiments ± SD; * p<0.01. (n.s.= non significant)

Appendix Figure S4

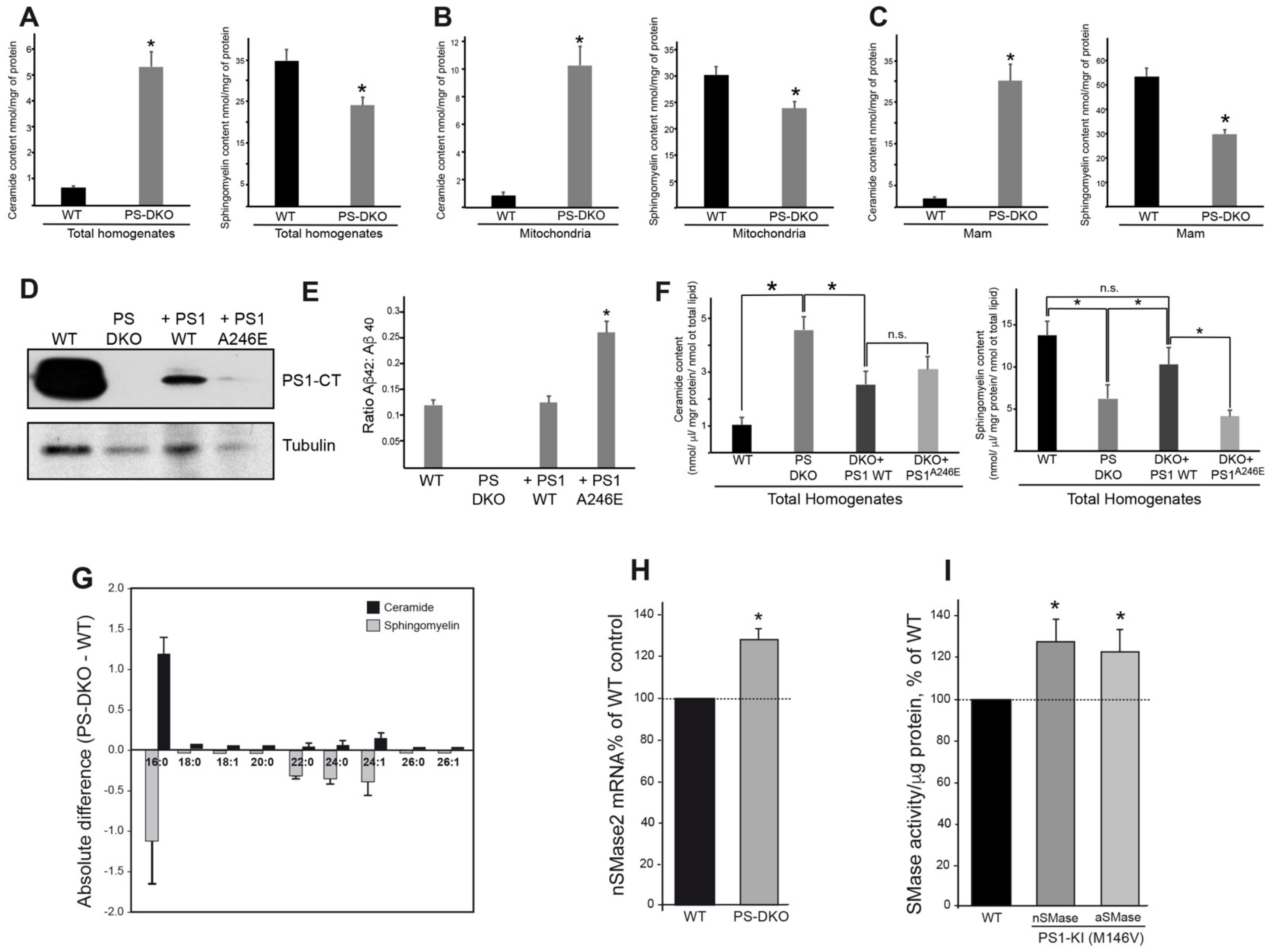

Appendix Figure S4 (Cont.)

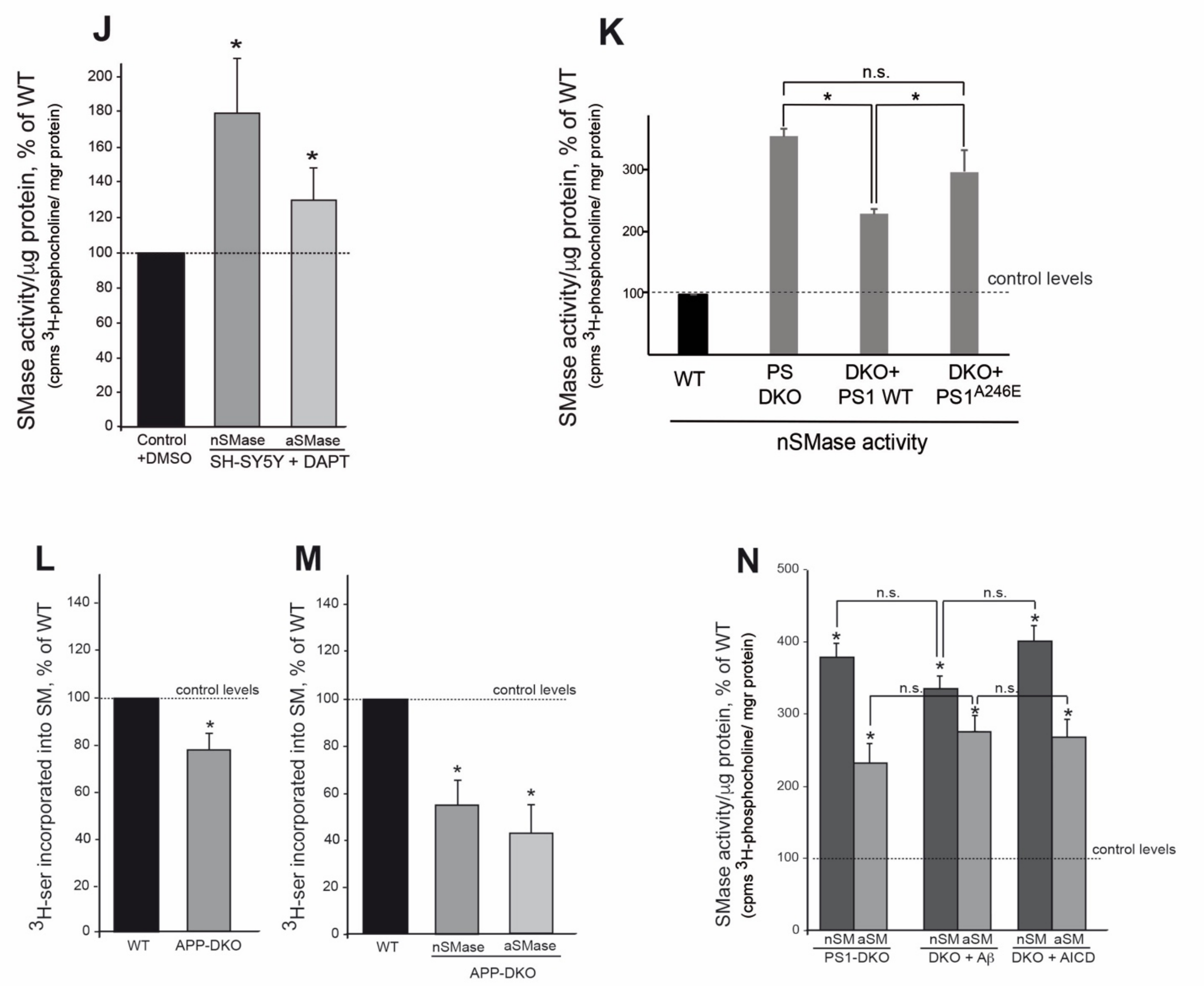

Appendix Figure S4. Acid (aSMase) and neutral (nSMase) sphingomyelinase activities in various cell types. (A-C) Ceramide and sphingomyelin levels in total homogenates (A), mitochondria (B), and MAM (C) from WT and PS-DKO cells, represented as nmol per μg of protein. **(D)** Western blot of WT and PS-DKO cell homogenates to reveal PS1 levels after transfection with the indicated plasmids. **(E)** Ratio of $A\beta_{42}$:$A\beta_{40}$ produced by cells shown in (D). Note the increase in the ratio of $A\beta_{42}$:$A\beta_{40}$ in cells transfected with mutant $PS1^{A246E}$. **(F)** Ceramide and sphingomyelin levels in PS-DKO cells transfected with the indicated plasmids. Note partial "rescue" of ceramide levels after transfection with WT-PS1, but not with $PS1^{A246E}$, plasmids. **(G)** Molecular species of ceramide and sphingomyelin in mitochondrial fractions from WT and PS-DKO MEFs. **(H)** Transcription of a gene encoding neutral sphingomyelinase 2 (nSMAse2; [*SMPD3*]), in WT and PS-DKO MEFs, by qRT-PCR. **(I)** Acid (aSMase) and neutral SMase (nSMAse) activities in brain from PS1-KI^{M146V} mice. Note increased SMase in the mutant cells compared to WT. **(J)** SMase activities in SH-SY5Y cells. Note significantly increased SMase activites following inhibition of γ-secretase activity (and increased C99) with DAPT. **(K)** Neutral SMase (nSMAse) activities in PS-DKO cells transfected with the indicated plasmids. Note decreased SMase in the mutant cells transfected with WT-PS1, but not with $PS1^{A246E}$, plasmids. **(L)** Sphingomyelin (SM) synthesis in APP-DKO MEFs. **(M)** Sphingomyelin hydrolysis or sphingomyelinase activities in APP-DKO MEFs (lacking C99). **(N)** Both aSMase (aSM) and nSMase (nSM), but particularly nSMase activity, are increased significantly in PS-DKO MEFs (containing C99 but lacking both Aβ and AICD) vs WT cells. These activities are essentially unchanged in the presence of either Aβ (monomers of $A\beta_{40}$ and $A\beta_{42}$ added to the medium at a ratio of $A\beta_{40}$:$A\beta_{42}$ of 10:1; 6 ng/ml total Aβ concentration) or AICD (expressed transiently from a plasmid). All experiments represent the average of n=5 independent experiments± S.D. * $p<0.05$; n.s., not significant.

Appendix Figure S5

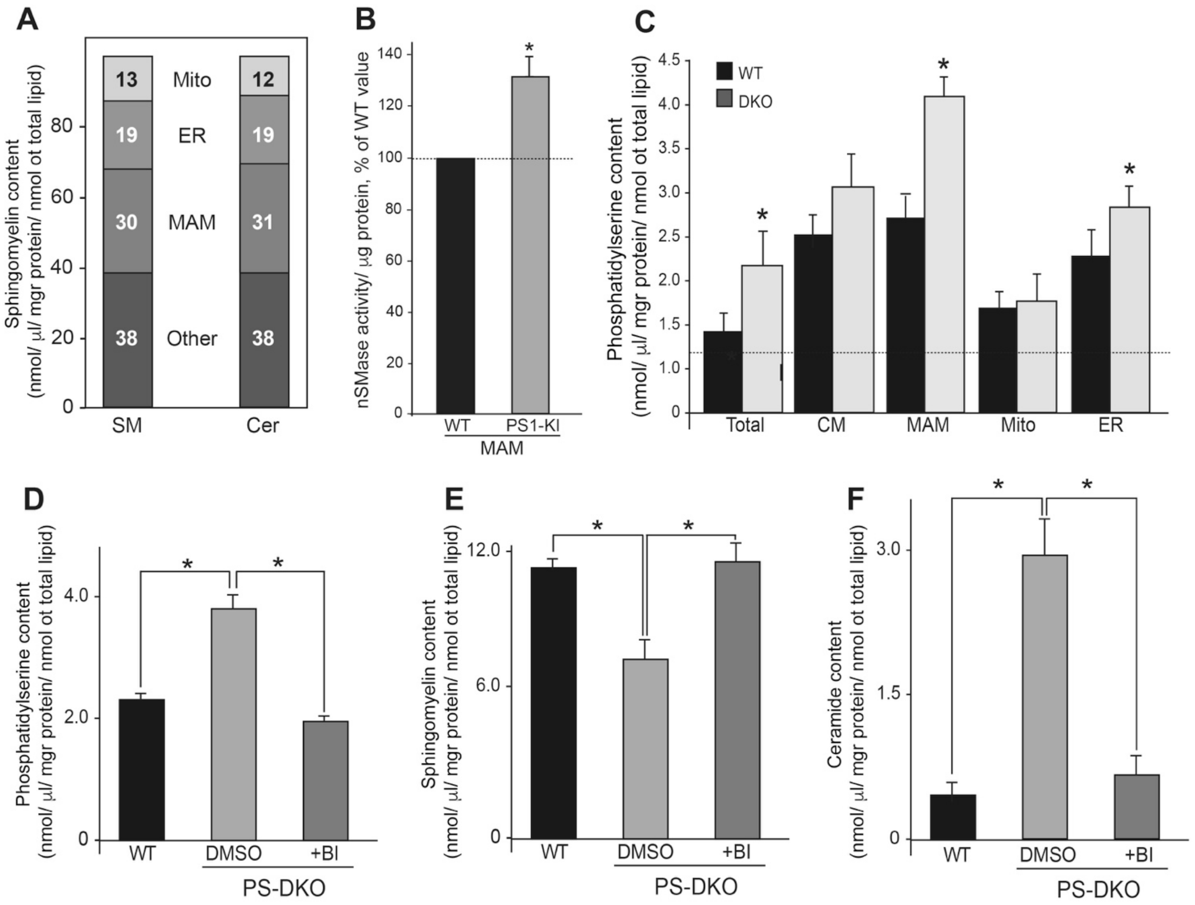

Appendix Figure S5. Analysis of sphingolipid metabolism in WT and PS-DKO MEFs. (A) Approximate distribution of steady-state levels of sphingomyelin (SM) and ceramide (Cer) in cell compartments in WT MEFs (numbers in boxes denote % of the total present in the indicated compartment). The "Other" value was derived from subtracting the MAM+ER+Mito values from those in the total homogenate. **(B)** Increased nSMase activity in MAM isolated from PS1-KI^{M146V} brain compared to WT brain (data represent the average of 4 independent experiments ±S.D. * p<0.05; n.s., not significant). **(C)** Phosphatidylserine (PtdSer) content in fractions isolated from WT and PS-DKO cells. Lipid values are represented as nmol normalized per unit of protein and total lipid extracted. Note the increase in PtdSer in MAM fractions (values represent the average of n=3 independent experiments ±S.D. * p<0.05; n.s., not significant). **(D)** PtdSer content in membranes isolated from WT, PS-DKO, and PS-DKO cells treated with BACE inhibitor. Note the decrease in PtdSer content in mutant cells after inhibiting the production of C99 with BI. Values represent the average of 3 independent experiments ±S.D. * p<0.05; n.s., not significant. **(E, F)** Sphingomyelin (E) and ceramide (F) levels in membranes isolated from WT, PS-DKO, and PS-DKO cells treated with a BACE1 inhibitor. Note that BI treatment rescues sphingolipid homeostasis in mutant cells. Values represent the average of n=3 independent experiments± S.D. * p<0.05; n.s., not significant.

Appendix Figure S6

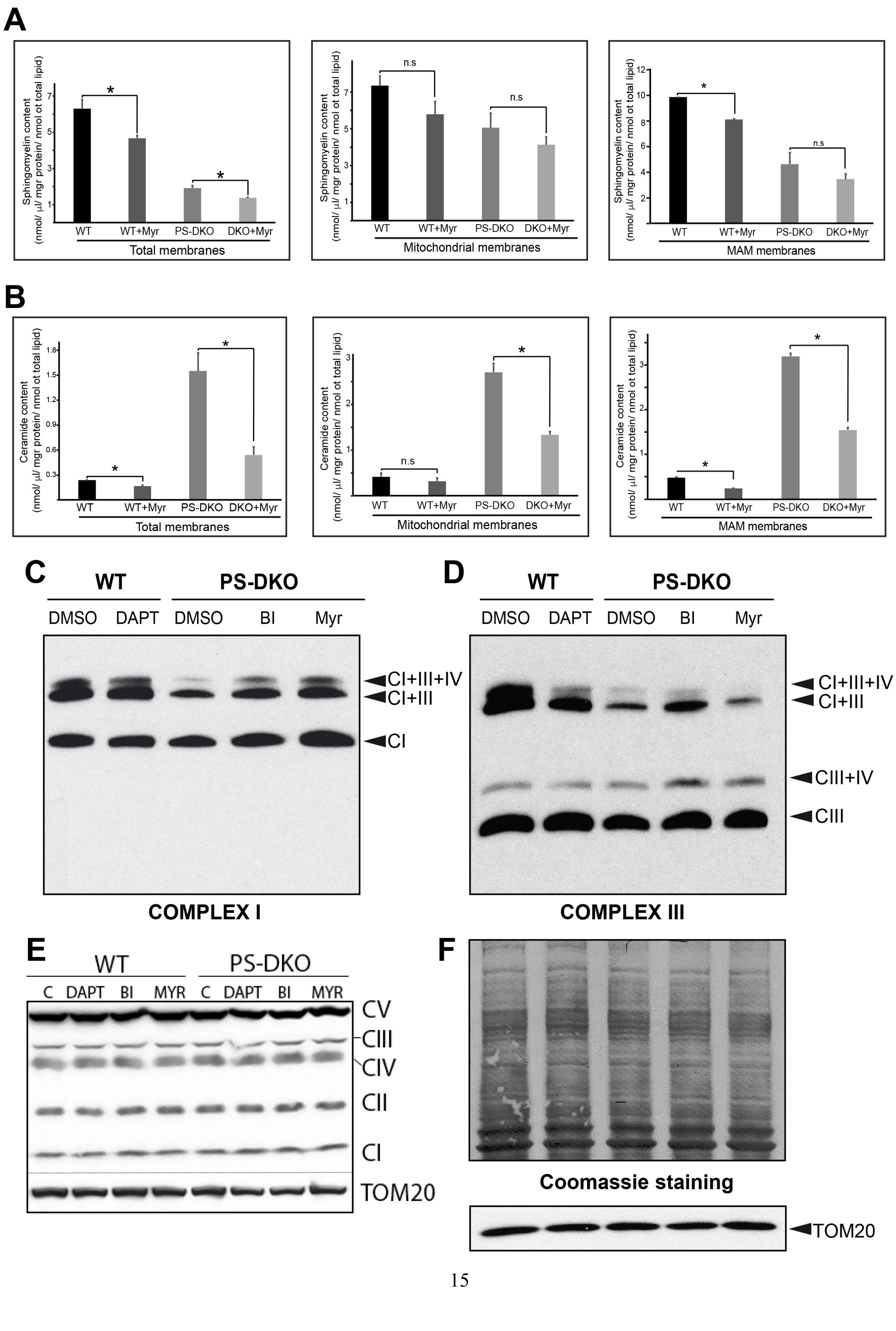

Appendix Figure S6. Supercomplex assembly is altered in γ-secretase deficient cells. (A, B) Sphingomyelin (A) and ceramide (B) levels in total homogenates, mitochondria, and MAM membranes from WT and PS-DKO cells untreated and treated with myriocin. Note significant reductions in sphingolipid levels after *de novo* synthesis of sphingolipids by myriocin. **(C, D)** Western blot of blue-native PAGE gels to detect complex I (C), and complex III (D) to reveal respiratory supercomplexes in mitochondria from WT and PS-DKO cells after the indicated treatments. Note that chemical or genetic inhibition of γ-secretase results in decreased supercomplex I+III+IV assembly. Inhibition of C99 production by BACE inhibitor (BI) or inhibition of ceramide production by myriocin (Myr) increases the assembly of supercomplexes I+III and I+III+IV as measured by anti-complex I antibodies, and of supercomplex III+IV as measured by anti-complex III antibodies. All values represent the average of n=3 independent experiments ± SD; * $p<0.05$; n.s., not significant. **(E)** Western blot of mitochondria isolated from WT and PS-DKO cells after the indicated treatments to reveal individual subunit complexes and TOM20. **(F)** As a loading control, the samples from (A) and (B) were analyzed by Coomasie staining or western blot to reveal TOM20 (a protein localized in the mitochondrial outer membrane). Dotted lines indicate a second western blot performed on the same membrame. Note that chemical or genetic inhibition of γ-secretase results in decreased supercomplex I+III+IV assembly. Inhibition of C99 production by BACE1 inhibitor (BI) or inhibition of ceramide production by myriocin (Myr) increases the assembly of supercomplexes I+III and I+III+IV as measured by anti-complex I antibody, and of supercomplex III+IV as measured by anti-complex III antibodies. All experiments represent the average of n=3 independent experiments± S.D. * $p<0.05$; n.s., not significant.

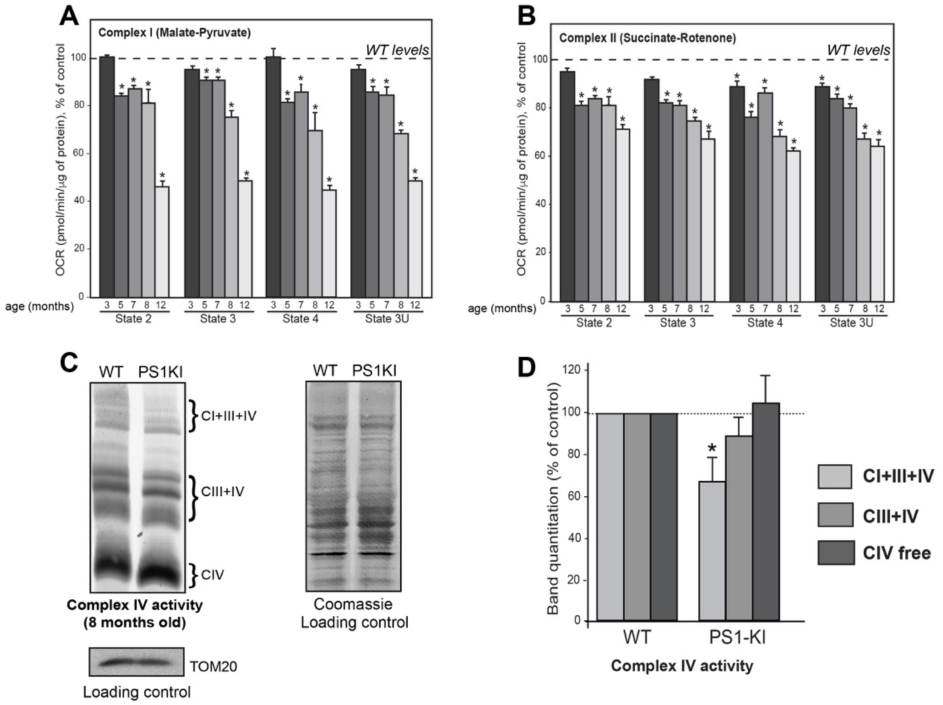

Appendix Figure. S7. **Mitochondrial dysfunction in PS1-KI^{M146V} mice. (A, B)** Oxygen consumption in mitochondria isolated from brain homogenates from PS1-KI^{M146V} mice analyzed at various ages (indicated in months) compared to that in the corresponding WT mice (set as 100%; dotted lines). In each assay, respiration is first measured at baseline; this is State 2 (i.e. initial respiration in the presence of added substrates [e.g. malate+pyruvate to measure complex I; succinate+rotenone to measure complex II]). ADP is then added; this is State 3 (i.e. maximum respiration in the presence of substrates). Oligomycin is then added to inhibit ATP synthase; this mimics State 4 (i.e. respiration after added ADP has been consumed). Finally, the uncoupler FCCP is added; this is State 3U (i.e. state 3 "uncoupled," [also called "noncoupled" respiration]. Note the steady decline in OCR that appears to accelerate at 8 months of age, correlating with **(C)** a decrease in supercomplexes activity, as measured by in-gel staining complex IV activity in mitochondria isolated from brain samples from 8 months old mice (upper left panel). As loading control, same membranes were analyzed by Coomassie staining (right panel), and the same samples were examined by western blot to detect Tom20 (bottom left panel). **(D)** Quantification

of bands from (C) by densitometry. All experiments represent the average of n=3 independent experiments± S.D. * $p<0.05$; n.s., not significant.
