# Supplementary material for: Increased localization of APP‐C99 in mitochondria‐associated ER membranes causes mitochondrial dysfunction in Alzheimer disease
Source: EMBO J. 2017 Oct 10;36(22):3356–71. doi: 10.15252/embj.201796797 (PMC5731665; doi:10.15252/embj.201796797)
Supplement: Supplementary file 2 — Source Data for Appendix [file EMBJ-36-3356-s003.zip › EMBOJ_96797_sourcedata_Fig_S2/96797_SD_figS2F.pdf]

Source data for Figure S2H

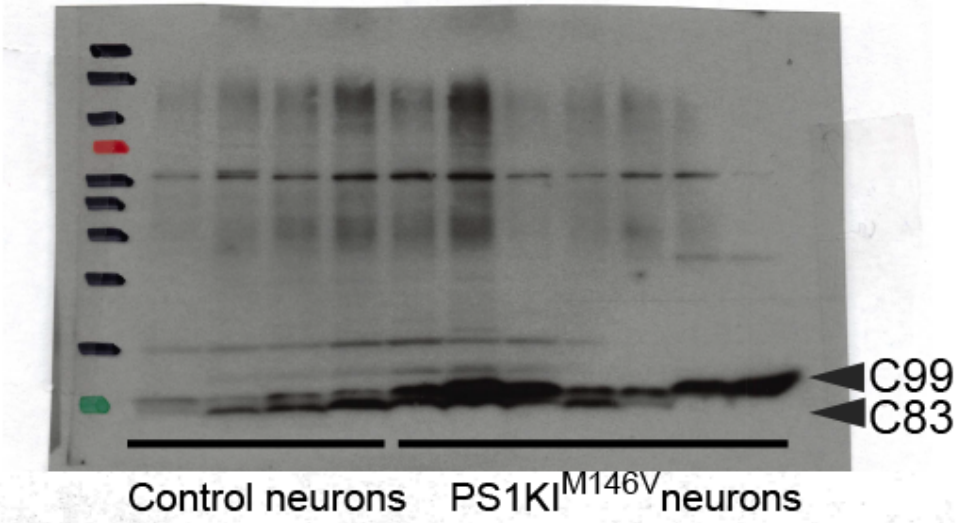

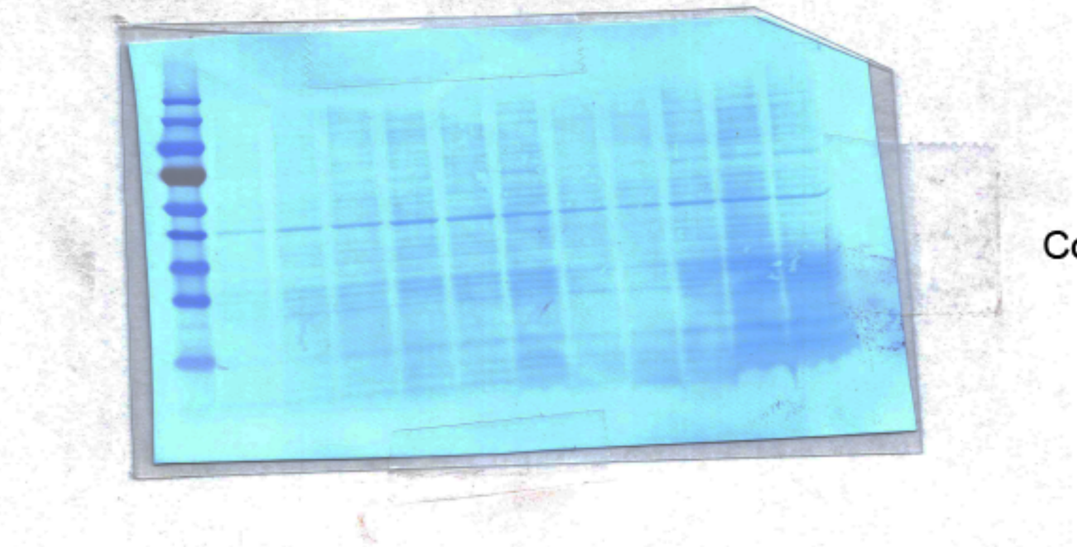

Coomassie staining

Source data for Appendix Figure S2F. Western blots of total homogenates of embryonic cortical neurons explanted from WT and PS1-KI^{M146V} mice show higher levels of C99 in mutant samples vs. controls (30 µg of protein per lane). Coomassie staining of membrane is below.
