# Supplementary material for: Increased localization of APP‐C99 in mitochondria‐associated ER membranes causes mitochondrial dysfunction in Alzheimer disease
Source: EMBO J. 2017 Oct 10;36(22):3356–71. doi: 10.15252/embj.201796797 (PMC5731665; doi:10.15252/embj.201796797)
Supplement: Supplementary file 2 — Source Data for Appendix [file EMBJ-36-3356-s003.zip › EMBOJ_96797_sourcedata_FigS6/96797_SD_figS6E.pdf]

Source data for Figure S6E

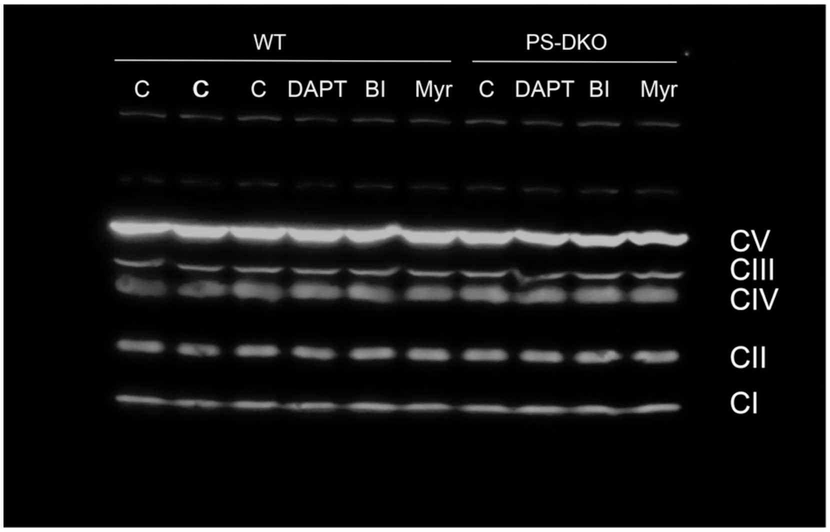

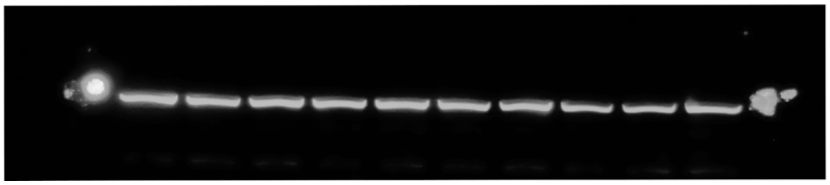

TOM20 as loading control

Source data for appendix figure S6E. Western blot of mitochondria isolated from WT and PS-DKO cells after the indicated treatments to reveal individual subunit complexes (Total OXPHOS WB antibody Cocktail (Abcam; ab110413) and TOM20 (Santa Cruz; sc-11415) as loading control (30μgr of protein per lane. Detected by chemiluminescent imaging system). (C=Control; DAPT= γ-secretase inhibitor; BI=BACE1 Inhibitor; Myr=Myriocin)
