# Supplementary figures and images for: Increased localization of APP‐C99 in mitochondria‐associated ER membranes causes mitochondrial dysfunction in Alzheimer disease
Source: EMBO J. 2017 Oct 10;36(22):3356–71. doi: 10.15252/embj.201796797 (PMC5731665; doi:10.15252/embj.201796797)

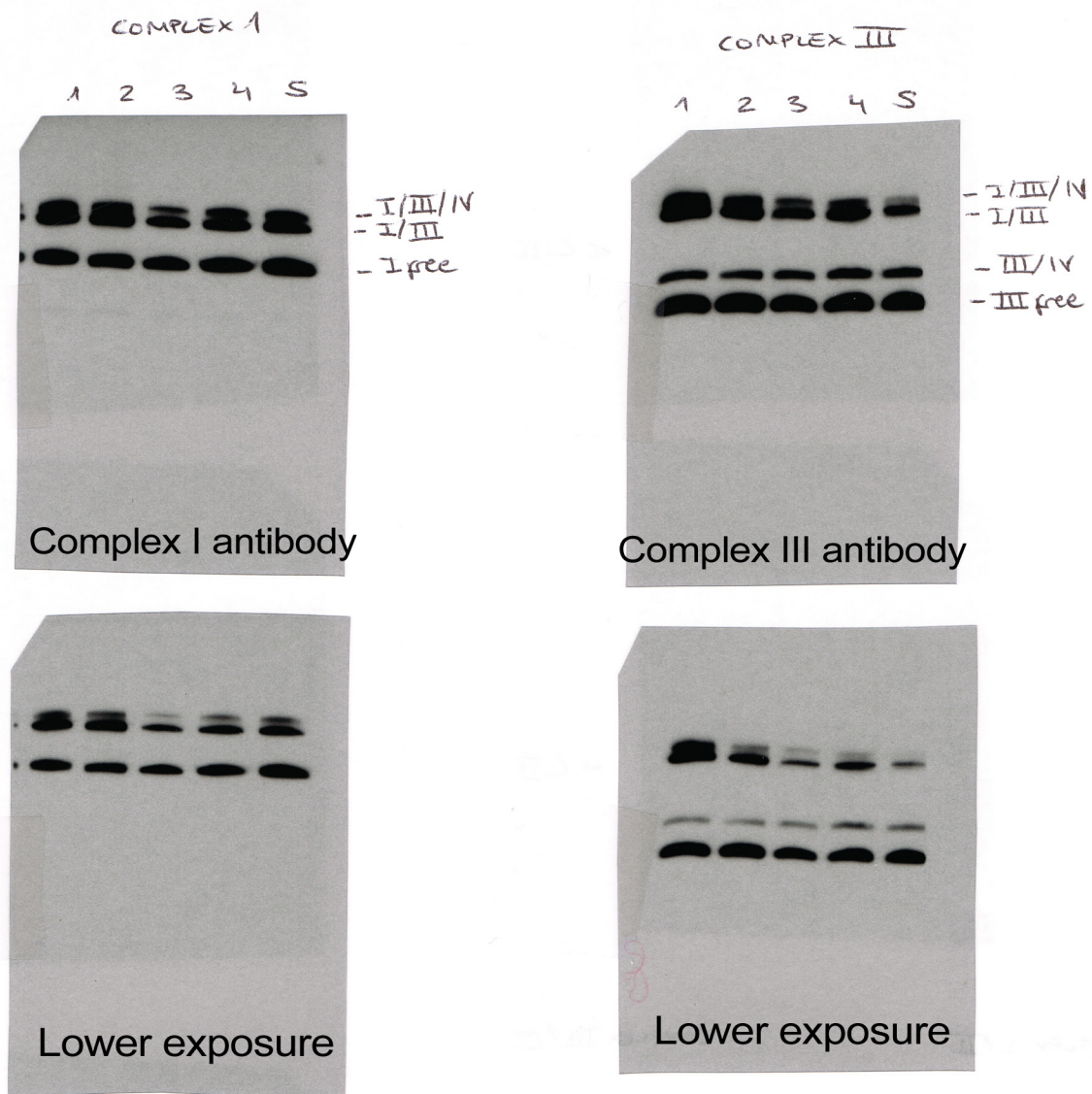

Western blot of same set of samples probed against Complex I and III

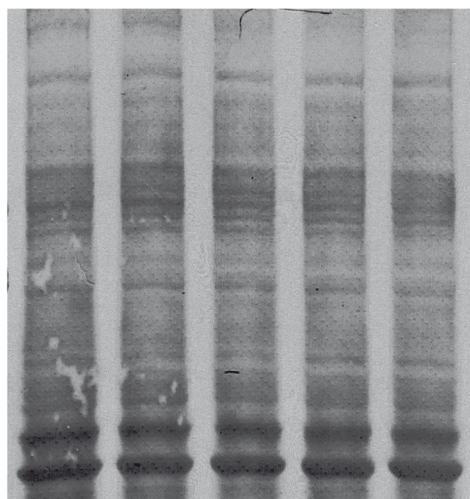

Coomassie staining

Supplement: Supplementary file 2 — Source Data for Appendix [file EMBJ-36-3356-s003.zip › EMBOJ_96797_sourcedata_FigS6/96797_SD_ S6C,D,F.pdf]
