# Supplementary material for: Increased localization of APP‐C99 in mitochondria‐associated ER membranes causes mitochondrial dysfunction in Alzheimer disease
Source: EMBO J. 2017 Oct 10;36(22):3356–71. doi: 10.15252/embj.201796797 (PMC5731665; doi:10.15252/embj.201796797)
Supplement: Supplementary file 4 — Source Data for Figure 6 [file EMBJ-36-3356-s002.pdf]

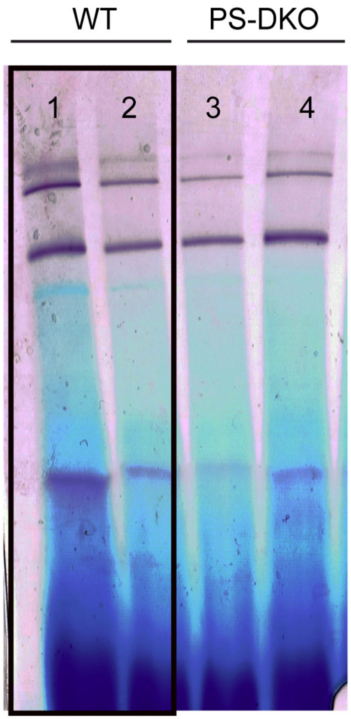

Complex I in gel activity

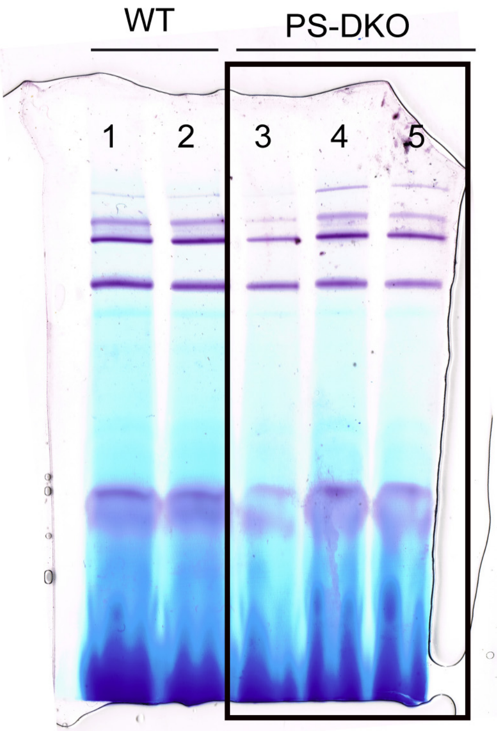

Complex I in gel activity

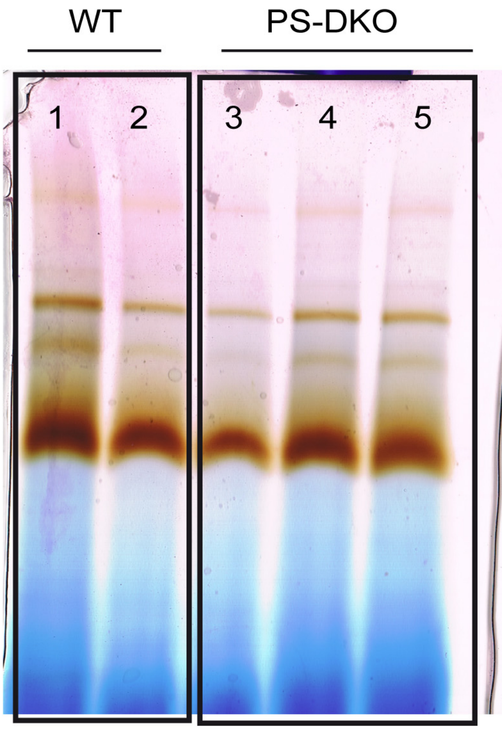

Complex IV in gel activity

1-WT cells with DMSO
2-WT cells with DAPT
3-PS-DKO cells with DMSO
4-PS-DKO cells + BACE1 inhibitor
5-PS-DKO cells + Myriocin

Squared lanes were used for the final figure

Source Data figure 6B and 6C
